# Oxygen Isotope Alterations during the Reduction of U_3_O_8_ to UO_2_ for Nuclear Forensics Applications

**DOI:** 10.1021/acsomega.3c03903

**Published:** 2023-09-05

**Authors:** Maor Assulin, Ruth Yam, Alina Grego-Shnaiderman, Yizhaq Eretz Kdosha, Noa Lulu-Bitton, Eyal Elish, Aldo Shemesh

**Affiliations:** † Department of Earth and Planetary Sciences, Weizmann Institute of Science, Rehovot 7610001, Israel; ‡ Analytical Chemistry Department, Nuclear Research Center Negev (NRCN), Beer Sheva 84190, Israel; § Materials Department, Nuclear Research Center Negev (NRCN), Beer Sheva 84190, Israel

## Abstract

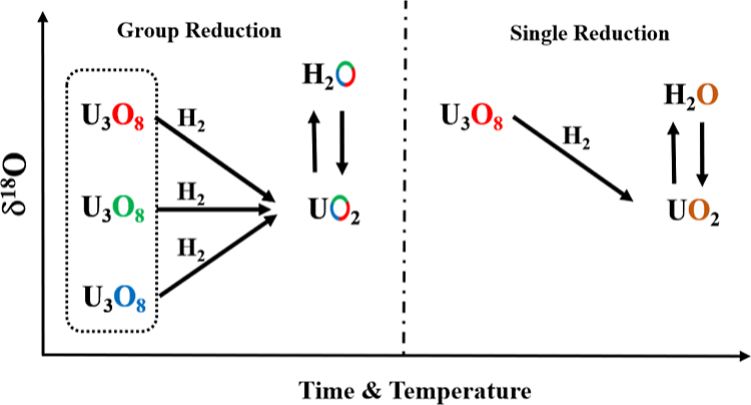

The fabrication of UO_2_ from U_3_O_8_ is an essential reaction in the nuclear fuel cycle. The oxygen isotope fractionation associated with this reaction has significant implications in the general field of nuclear forensics. Hence, the oxygen isotope fractionation during the reduction of U_3_O_8_ to UO_2_ was determined in the temperature range of 500–700 °C and for a duration of 2 to 6 h under a high-purity H_2_ atmosphere. Three U_3_O_8_ samples, possessing a different oxygen isotopic composition, were used to investigate key parameters involved with the fractionation during the reduction process. All UO_2_ products did not maintain the original isotope composition of the starting U_3_O_8_ under all conditions. The results show that the system UO_2_–H_2_O attains isotope equilibrium at 600 °C, provided the reduction process lasts at least 4 h or more. At 600 °C, UO_2_ was isotopically depleted by 2.89 ± 0.82‰ compared to the U_3_O_8_ from which it was produced. We find that the H_2_O formed during the reduction plays a major role in determining the final δ^18^O of UO_2_ prepared from U_3_O_8._ The isotope equilibrium of the system UO_2_–H_2_O at 600 °C was calculated, indicating that δ^18^O of the H_2_O was enriched by about 11‰ relative to the UO_2_ due to the uranium mass effect. These findings could potentially have important implications for nuclear forensics, as they provide a new method for determining the history of UO_2_ samples and tracing back their production process.

## 1 Introduction

Uranium dioxide pellets (UO_2_) are a major end-product in the nuclear fuel cycle, beginning with uranium ores. The illicit trafficking of uranium from one of the production stages has occurred in the past, attracting significant scientific attention in nuclear forensics. Therefore, an ongoing effort has been dedicated to developing new analytical techniques, focusing on finding novel signatures to allow improved and credible attribution of uranium compounds found outside of regulatory control.

UO_2_ in the reduction process of U_3_O_8_ to UO_2_ is usually applied at high temperatures (above 500 °C) under a reducing environment (e.g., pure H_2_ or a mixture of H_2_/Ar),^1^ and the chemical reaction is given in eq 1.
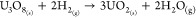
1

The mechanism and kinetic model for this reaction at 510 °C identified the rate-limiting step as the desorption of water vapors from the external surface of the U_3_O_8_ particles while conversion proceeds.^2^ The study of the diffusion of oxygen in the natural uraninite–H_2_O at a temperature range of 50–700 °C suggested an initial extremely fast-path diffusion mechanism that overprints the oxygen isotopic composition of the entire crystal, independent of temperature, and a slower volume-diffusive mechanism dominated by defect clusters.^3^ Volume diffusion was also considered the primary process controlling oxygen diffusion in polycrystalline and single-crystalline UO_2_ in the temperature range of 605–750 °C.^4^ Depth profiling beneath the UO_2_ pellet surface at room temperature indicates oxygen diffusion into the UO_2_ lattice, while water species diffusion occurs along grain boundaries, behaving as high-diffusivity paths.^5^

The use of the oxygen isotopic composition (^18^O/^16^O—expressed in δ notation as δ^18^O vs VSMOW) as a signature for geolocation^6−8,11−13^ and the process history^9,10,15−18^ of uranium oxides for nuclear forensics applications^14^ have gained much interest in recent years. The δ^18^O variation of naturally occurring uranium ore minerals is in the range of −32 to +11‰.^8^ However, the correlation with local meteoric water is still in dispute, partially due to the lack of data on the fluid isotopic composition from which the minerals were precipitated or on post-formation isotope alteration of the original uraninite (UO_2_).^4−6^ On the other hand, it has been documented that uranium oxides of different geographic origins have significantly different ^18^O/^16^O ratios, which correlate with local rainwater isotopic composition.^6^

The comparison of several oxygen extraction methods from UO_2_, U_3_O_8_, and UO_3_ compounds has revealed that different amounts of water molecules present in the uranium oxides must be removed, as they interfere with the isotope measurement^11^ and exchange reactions with humid atmospheres, affecting the oxygen fractionation.^15^ Klosterman et al.^15^ synthesized U_3_O_8_ from uranium peroxide at 300–1000 °C, reporting fractionation of about −22‰ up to about −5‰, respectively, between the oxide and atmospheric oxygen with a retrograde isotope effect.^16^ The exposure of U_3_O_8_ and UO_2_ to humidity in an oxidizing atmosphere showed that hydrated uranium oxide grows as a secondary mineral (metaschoepite) on aged U_3_O_8_ and UO_2._ It was suggested that δ^18^O values of the metaschoepite hydration water are likely to reflect those of the water vapor to which the sample was exposed.^12,13^ Thus, the oxygen isotopes of metaschoepite mineral hydration water may retain information on the mineral formation location.

Hattori and Halas^19^ theoretically investigated the fractionation factors α (defined as the isotopic ratio between two compounds) in the systems UO_2_–H_2_O and UO_3_–H_2_O over a temperature range of 0–1000 °C, finding that uraninite is consistently depleted with ^18^O compared to its associated H_2_O across this temperature range. Yong-fei^20^ developed a theoretical model to calculate the oxygen isotopic fractionation factors of metal oxides, including uraninite, over the same temperature range. However, the two models^19,20^ produced different results, with a difference of approximately 10‰ in 1000 ln α (UO_2_–water) at temperatures up to 300 °C and a difference of ∼2‰ at higher temperatures between 300 and 600 °C. Fayek and Kyser^9^ found fractionation factors similar to those reported by Hattori and Halas^19^ at high temperatures but closer to those predicted by Yong-fei^20^ at low temperatures.

This study focuses on the oxygen isotope change resulting from the manufacturing processes of UO_2_ from U_3_O_8_. UO_2_ powders were synthesized in the temperature range of 500–700 °C for 2–6 h under high purity H_2_ to follow the process of isotope partition among U_3_O_8_ as the source material, UO_2_, and H_2_O as products of the reduction reaction.

## 2 Materials and Methods

Three U_3_O_8_ samples with different oxygen isotopic compositions were used as starting materials for this study. Two were synthesized from uranyl nitrate hydrate (UNH). U_3_O_8_-I has a δ^18^O value of 10.68 ± 0.27‰, and U_3_O_8_-II has a δ^18^O value of 8.52 ±0.22‰. These two materials were synthesized by changing the cooling rate to achieve the difference in their δ^18^O values.^17^ The third sample, U_3_O_8_-III, has a δ^18^O value of 4.75 ± 0.47‰ and is a natural uranium commercial U_3_O_8_ material purchased from CETAMA, France (commercially known as “Chanterelle”), usually used as a calibration standard for elemental impurities in uranium.

The samples (∼100 mg each) were weighed in an alumina crucible and placed at room temperature in a stainless-steel furnace before applying a vacuum (∼10^–5^ Torr) for 12 h. The samples were calcined for 2, 4, and 6 h at a temperature range of 500–700 °C under high purity (99.999%) H_2_ 1 atm, followed by cooling the reactor to room temperature via shutting down the furnace. The three U_3_O_8_ samples were placed together in the reactor (the UO_2_ products are marked as samples 1UO_2_–27UO_2_ in Table 1). One set of experiments was conducted where each U_3_O_8_ sample was placed alone at 600 °C for 4 h (samples 31UO_2_–33UO_2_ were those placed separately; Table 1). Table 1 presents all experiments’ temperatures, samples, and experimental conditions calcined time.

**Table 1:** δ^18^O (in ‰ Relative to VSMOW) Values of U_3_O_8_ and UO_2_ Samples Prepared at Different Temperatures

sample	δ^18^O (‰ VSMOW)	SD (‰)	temp. (°C)	time (h)	source
U_3_O_8_-I^a^	10.68	0.27			UNH
U_3_O_8_-II^a^	8.52	0.22			UNH
U_3_O_8_-III^a^	4.75	0.47			commercial
1UO_2_	4.31	0.59	500	2	U_3_O_8_-I
2UO_2_	5.11	0.59			U_3_O_8_-II
3UO_2_	5.78	0.37			U_3_O_8_-III
4UO_2_	7.72	0.57	600	2	U_3_O_8_-I
5UO_2_	7.02	0.69			U_3_O_8_-II
6UO_2_	5.09	0.34			U_3_O_8_-III
7UO_2_	4.82	0.37	700	2	U_3_O_8_-I
8UO_2_	5.34	0.33			U_3_O_8_-II
9UO_2_	7.08	0.37			U_3_O_8_-III
10UO_2_	5.67	0.21	500	4	U_3_O_8_-I
11UO_2_	4.63	0.38			U_3_O_8_-II
12UO_2_	4.54	0.31			U_3_O_8_-III
13UO_2_	5.34	0.34	600	4	U_3_O_8_-I
14UO_2_	5.54	0.27			U_3_O_8_-II
15UO_2_	5.55	0.13			U_3_O_8_-III
16UO_2_	7.17	0.19	700	4	U_3_O_8_-I
17UO_2_	6.37	0.04			U_3_O_8_-II
18UO_2_	7.16	0.11			U_3_O_8_-III
19UO_2_	5.36	0.20	500	6	U_3_O_8_-I
20UO_2_	5.30	0.09			U_3_O_8_-II
21UO_2_	5.07	0.15			U_3_O_8_-III
22UO_2_	6.21	0.44	600	6	U_3_O_8_-I
23UO_2_	5.86	0.45			U_3_O_8_-II
24UO_2_	6.21	0.30			U_3_O_8_-III
25UO_2_	4.94	0.02	700	6	U_3_O_8_-I
26UO_2_	4.39	0.44			U_3_O_8_-II
27UO_2_	4.62	0.35			U_3_O_8_-III
31UO_2_	8.64	0.77	600	4	U_3_O_8_-I
32UO_2_	4.84	0.43	600	4	U_3_O_8_-II
33UO_2_	1.81	0.50	600	4	U_3_O_8_-III
NBS-28^b^	9.51 ± 0.42				

a The highlighted row corresponds to the δ^18^O values of the starting U_3_O_8_ samples.

b NBS-28 has an assigned isotope value of 9.58 ± 0.09‰ as an international standard.^22^

XRD analysis (Rigaku, Ultima III) was performed on samples weighing several milligrams under an atmospheric environment by continuous scanning at 40 kV/40 mA in the range of 10–80° at a rate of 2°/min. The analyzed samples were the starting materials (U_3_O_8_-I, U_3_O_8_-II, and U_3_O_8_-III) and those prepared at 500 °C; 2 h, 500 °C; 4 h, and 600 °C; 2 h.

Oxygen isotopic analyses of the U_3_O_8_ and UO_2_ samples were conducted using an isotope ratio gas-chromatography-mass spectrometer (irmGCMS, Thermo Scientific Delta Plus Advantage) and an IR CO_2_ laser (10.6 μm, New Wave Research 25 W). The method is described in detail elsewhere.^14,17,18,21^ Briefly, U_3_O_8_ samples (1026–1651 μg), UO_2_ samples (1240–1700 μg), and SiO_2_ samples (NBS-28, standard material for quality check and calibration; 260–450 μg) were placed in nickel cups in a stainless-steel chamber and heated overnight at 80 °C under a high vacuum. Pre-fluorination was performed thrice for the entire cell with 80 Torr of BrF_5_. Samples were reacted by laser heating in a 90 Torr of BrF_5_ atmosphere. The liberated oxygen was purified by liquid nitrogen traps, concentrated on a 5 Å molecular sieve, cooled in liquid nitrogen, and transferred to the mass spectrometer through a gas chromatograph column for isotope measurement in a continuous flow mode. The international SiO_2_ standard NBS-28 (δ^18^O = 9.58‰)^22^ was used for consistency and calibration in each batch. The measured values are expressed in δ-notation in peril, relative to Vienna Standard Mean Ocean Water (VSMOW). The long-term standard deviation (SD) for NBS-28 was 0.42‰. All the samples were run at least in triplicate, and the SD is reported for each sample.

## 3 Results

### 3.1 XRD

**Figure 1:**
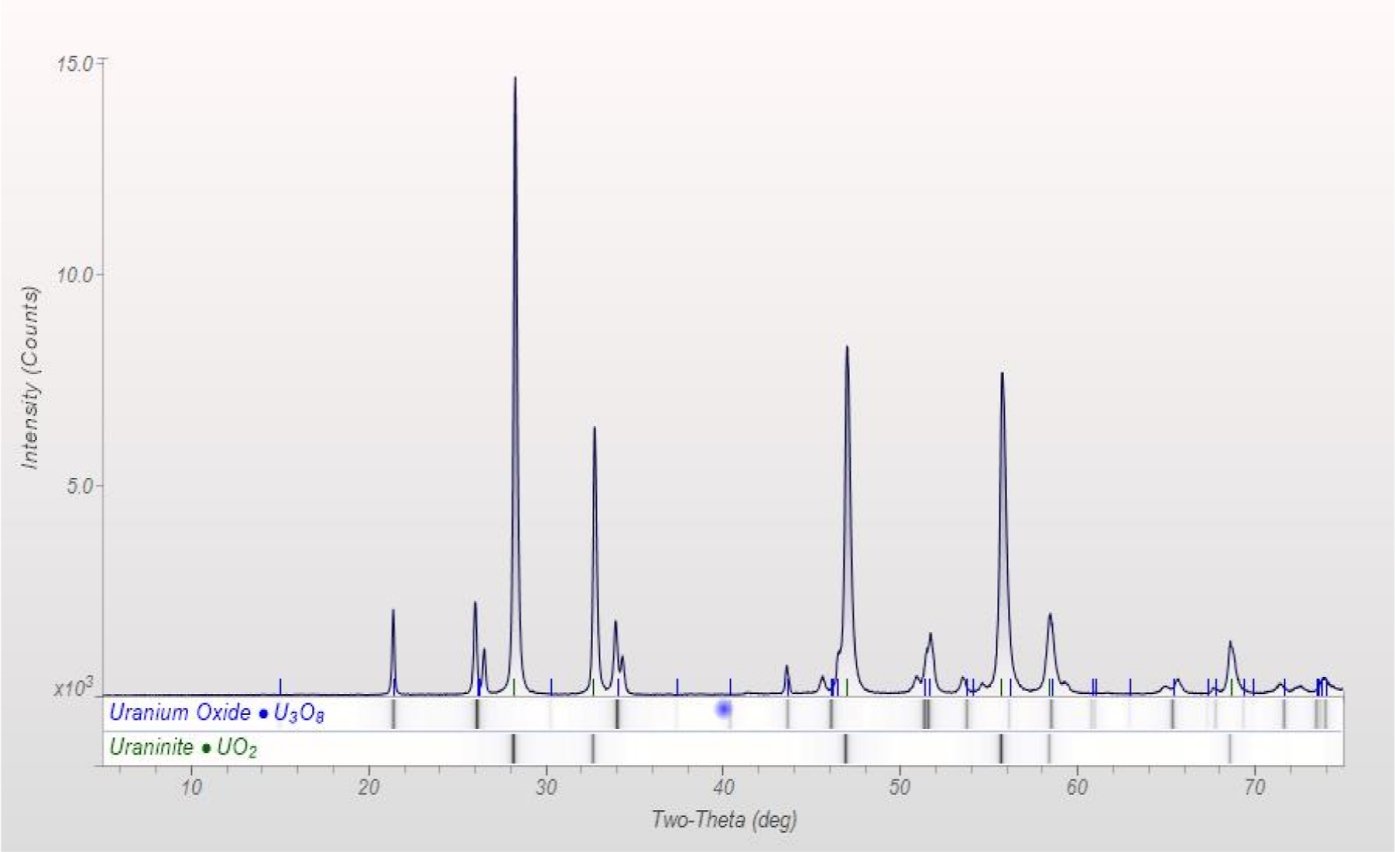
XRD diffractograms of the samples prepared at 500 °C for 2 h showing a mixed phase of UO_2_ and U_3_O_8_.

**Figure 2:**
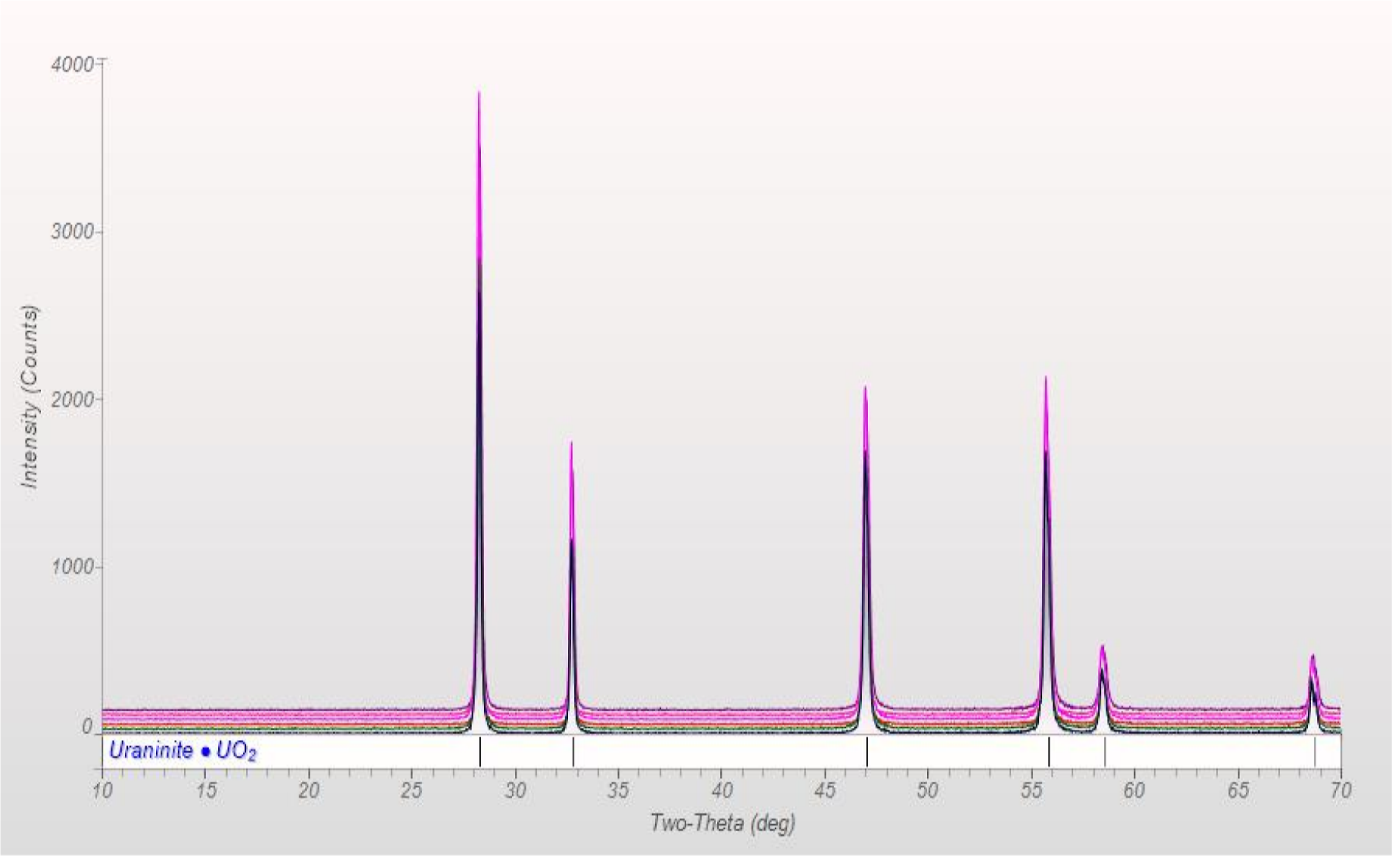
XRD diffractograms of the samples prepared at ≥500 °C for 4 h showing a complete reduction to a single UO_2_ phase.

The XRD diffractograms of the samples prepared at 500 °C for 2 h present a mix of UO_2_ and U_3_O_8_ (Figure 1) phases, while samples prepared at 500 °C for 4 h and 600 °C for 2 h present a single UO_2_ phase (Figure 2).

### 3.2 δ^18^O of U_3_O_8_

**Figure 3:**
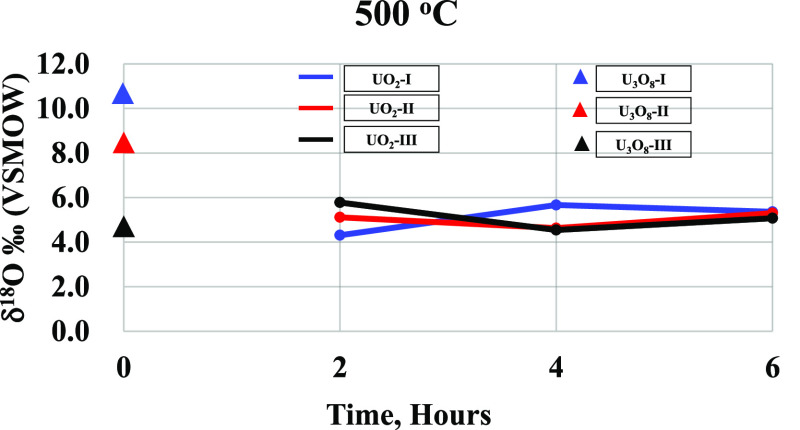
δ^18^O (in ‰ relative to VSMOW) values for the starting materials at room temperature (U_3_O_8_ at 25 °C) and UO_2_ samples at 500 °C for 2 to 6 h. The SD is within the symbol size.

**Figure 4:**
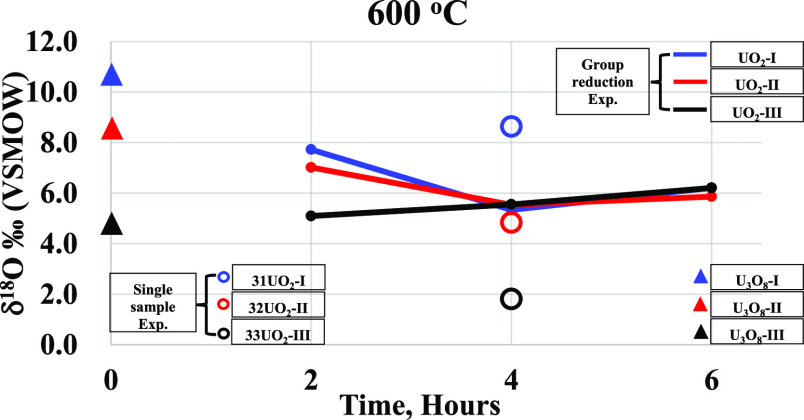
δ^18^O (in ‰ relative to VSMOW) values for the starting materials (red, blue and black triangles) at room temperature (U_3_O_8_ at 25 °C) and UO_2_ samples at 600 °C for 2–6 h from the single sample experiments (red, blue, and black empty circles) and from the group reduction experiments (red, blue, and black lines). The SD is within the symbol size.

**Figure 5:**
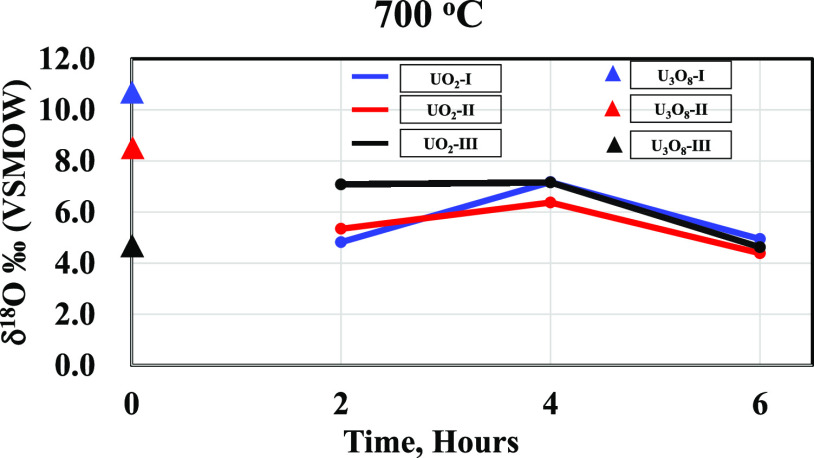
δ^18^O (in ‰ relative to VSMOW) values for the starting materials at room temperature (U_3_O_8_ at 25 °C) and UO_2_ samples at 700 °C for 2–6 h.

The average δ^18^O values of the starting materials (U_3_O_8_) and the UO_2_ samples (3–5 measurements for each sample), prepared at the temperatures range of 500–700 °C for 2–6 h, and of NBS-28 samples, are presented in Table 1. The standard deviations (SD) are within the size of the symbols in the figure. The δ^18^O values of UO_2_ samples as a function of calcination time are plotted for each calcination temperature in Figures 3–5. The δ^18^O precision of the UO_2_ is identical to the routinely measured NBS-28 standards.

The results (Table 1) will be evaluated according to the two groups of experiments. The first is the reduction of a single U_3_O_8_ sample in the reduction furnace at 600 °C for 4 h, and the second is where the three original U_3_O_8_ samples were reacted together at different times and different temperatures. δ^18^O of UO_2_ obtained from U_3_O_8_ of the first group is lower by 2.1, 3.7, and 3‰ than the original values for samples 31UO_2_, 32UO_2_, and 33UO_2_, respectively. The results from the second group show a general trend of conversion to a common isotope value, which is unique to each temperature. The difference among samples is larger at a reaction time of 2 h and decreases with increasing reaction time. The samples prepared from U_3_O_8_-I and U_3_O_8_-II did not retain the original δ^18^O values and showed a similar trend toward depleted values, while the UO_2_ prepared from U_3_O_8_-III presented an opposite trend toward a small enrichment. At a calcination temperature of 500 °C for 4 h (Figure 4), none of the samples retained the original δ^18^O values, showing similar trends toward depletion. At 600 and 700 °C, the δ^18^O values obtained for all three samples showed enriched and similar end values. The δ^18^O values of the samples that were reduced at 500, 600, and 700 °C for 6 h (Figure 5) exhibited identical values within the standard deviations, with U_3_O_8_-I and U_3_O_8_-II being 6 and 4‰ depleted, respectively, from the original U_3_O_8_ values. The δ^18^O value of sample U_3_O_8_-III converged to the same value as that of U_3_O_8_-I and U_3_O_8_-II, which was very close to its original value.

## 4 Discussions

The conversion of U_3_O_8_ to UO_2_ under a H_2_ atmosphere involves releasing water from the U_3_O_8_ (eq 1). The conversion process was studied by Pijolat et al.^2^ and Alfaro et al.^1^ in an open system at high temperatures under variable flow and variable mixtures of gases, H_2_/N_2_/He, and water vapor. Full conversion to UO_2_ within several minutes up to 1 h was achieved under flow conditions. In contrast to the open system flow experimental configurations, our reduction of U_3_O_8_ was carried out within a closed system, maintaining a constant pressure of high purity H_2_ and a significant molar excess of H_2_ (2.14 mmol relative to the U_3_O_8_ 0.11 mmol) at temperatures of 500, 600, and 700 °C. We attribute the partial conversion of U_3_O_8_ to UO_2_ at 500 °C for 2 h to the difference between the closed- versus open-flow experimental setups.

UO_2_ does not retain the original δ^18^O value of the U_3_O_8_ starting materials at all temperatures and reaction times. Nevertheless, at high temperatures (≥600 °C) and longer reaction times (≥4 h), δ^18^O converges to a common value when the three starting materials are reacted together and fully converted, indicating the establishment of an isotope equilibrium between the reaction products, UO_2_ and H_2_O. Thus, the similar δ^18^O values obtained from the three different original materials represent an isotope equilibrium with the generated mixture of the three waters, and therefore, a UO_2_–H_2_O fractionation factor cannot be deduced from combined material experiments.

A different set of reduction experiments, where samples 31UO_2_–33UO_2_ were placed separately at 600 °C for 4 h, was conducted to resolve this problem. It allows calculating the oxygen isotopic composition of the formed water through mass balance considerations and the fractionation factor between U_3_O_8_ and UO_2_. The isotopic composition of the water will be calculated in the next section.

### 4.1 Oxygen Isotopic Composition of H_2_O

The oxygen isotope mass balance can be used to calculate the oxygen isotopic composition of the formed water molecule, considering a closed system case where a single sample is converted from U_3_O_8_ to UO_2_ under an H_2_ atmosphere (samples 31UO_2_, 32UO_2_, and 33UO_2_). The isotope mass balance equation is given by
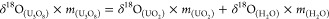
2where *m* represents the mass of the material indicated in the parenthesis, and δ^18^O is its oxygen isotopic composition. The values of the parameters δ^18^O_(U_3_O_8_)_, *m*_(U_3_O_8_)_, and δ^18^O_(UO_2_)_ in eq 2 are measured, while the values of the parameters *m*_(UO_2_)_ and *m*_(H_2_O)_ are calculated to determine δ^18^O_(H_2_O)_. Table 2 summarizes the first step of calculations, which yields the mass of the produced UO_2_ and formed H_2_O. The calculations are based on the following assumptions: (1) U_3_O_8_ and UO_2_ are composed of a single phase, (2) U_3_O_8_ consists of 84.8% uranium and 15.2% oxygen by mass, and UO_2_ consists of 88.1% uranium and 11.9% oxygen by mass, and (3) during the reduction reaction, U_3_O_8_ is fully converted to UO_2_ and the amount of uranium remains constant. Hence, under these assumptions, 1 mg of U_3_O_8_ produces 0.9620 mg of UO_2_ and 0.0428 mg of H_2_O.

**Table 2:** Calculation of the UO_2_ and Formed H_2_O Mass

sample	U_3_O_8_				UO_2_			H_2_O	
	initial mass (mg)	mass of U (mg)	mass of O (mg)	δ^18^O (‰ VSMOW)	mass of O (mg)	mass of UO_2_ (mg)	δ^18^O (‰ VSMOW)	Mass of O (mg)	mass of H_2_O (mg)
31UO_2_	99	84.0	15.05	10.68	11.3	95.2	8.64	3.8	4.2
32UO_2_	100	84.8	15.2	8.52	11.4	96.2	4.84	3.8	4.3
33UO_2_	100	84.8	15.2	4.75	11.4	96.2	1.81	3.8	4.3

Rearranging eq 2, the calculated δ^18^O_(H_2_O)_ for each sample is given by
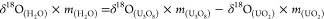
3

The calculated δ^18^O_(H_2_O)_ values for samples 31UO_2_, 32UO_2_, and 33UO_2_ are 16.81, 19.57, and 13.56‰, respectively.

We further apply the mass balance calculation to a mixture of 3 samples to test our assumptions and calculations. Samples 13UO_2_, 14UO_2_, and 15UO_2_ were placed together in the reactor and prepared under the same temperature, time, and pressure conditions as the single sample reaction above (Table 2). The final δ^18^O_(H_2_O)_, which results from mixing three different waters, is calculated using eq 4, which originates from solving a set of 3 separate mass balance equations. Table 3 summarizes the calculated δ^18^O of the total formed H_2_O.
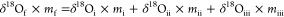
4where: δ^18^O_f_—δ^18^O of the final mixture of the formed water molecules (‰). *m*_f_—the mass of the total formed water molecules (mg). δ^18^O_i_/δ^18^O_iii_/δ^18^O_iii_—δ^18^O of U_3_O_8_-I, U_3_O_8_-II, and U_3_O_8_-III, respectively (‰). *m*_i_/*m*_ii_/*m*_iii_—mass of U_3_O_8_-I, U_3_O_8_-II, and U_3_O_8_-III, respectively (mg).

**Table 3:** Calculated δ^18^O of the Total Formed H_2_O

	U_3_O_8_				UO_2_			H_2_O			
sample	initial mass (mg)	mass of U (mg)	mass of O (mg)	δ^18^O (‰ VSMOW)U_3_O_8_	mass of O (mg)	mass of UO_2_ (mg)	δ^18^O (‰ VSMOW)UO_2_	mass of O (mg)	mass of H_2_O (mg)	total mass of H_2_O (mg)	δ^18^O of total H_2_O (‰)
13UO_2_	123	104.3	18.7	10.68	14.0	118.3	5.34	4.7	5.3	14.9	16.64
14UO_2_	109	92.4	16.6	8.52	12.4	104.9	5.54	4.1	4.7		
15UO_2_	117	99.2	17.8	4.75	13.3	112.6	5.55	4.4	5.0		

The result, 16.64‰, agrees well if we consider this water to be a mixture of the previously calculated waters of samples 31UO_2_, 32UO_2_, and 33UO_2_. Further, based on eq 4, in a closed system containing water molecules with an average δ^18^O value of 16.64 ± 3.01‰, the theoretical expected δ^18^O of the UO_2_ samples is 5.14‰. The measured δ^18^O values of samples 13UO_2_, 14UO_2_, and 15UO_2_ were similar: 5.34 ± 0.34, 5.54 ± 0.27, and 5.55 ± 0.13‰, respectively. We conclude that the final δ^18^O of UO_2_ prepared from U_3_O_8_ is determined by isotope equilibrium between the products of the reduction reaction, UO_2_, and H_2_O.

### 4.2 Isotope Fractionation

The XRD measurements show that the initial U_3_O_8_ was fully converted to UO_2_ and H_2_O in our analytical setup, except for the 500 °C and 2 h reaction. Therefore, the fractionation factor between U_3_O_8_ and UO_2_ cannot be calculated because these two phases were not sampled along the reaction path and do not co-exist in isotope equilibrium at the final stage. It is interesting to note that although the two phases, U_3_O_8_ and UO_2_ co-exist, the final δ^18^O converges to a similar value. This may indicate that the isotope exchange between U_3_O_8_, UO_2_, and H_2_O is faster than the reduction reaction.

However, the difference in δ^18^O (Δ^18^O) between UO_2_ and H_2_O can be calculated (eq F035).
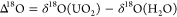
F035

Equation F035 was applied to the average δ^18^O values of samples 13UO_2_, 14UO_2_, and 15UO_2_ (5.48 ± 0.12‰) and the average δ^18^O value of the calculated formed water (16.64 ± 3.01‰). The calculated Δ^18^O at 600 °C in this work is −11‰. This result, where the water is isotopically enriched relative to the solid, can be explained by the uranium “mass effect” (i.e., the tendency of uranium to hold the lighter atoms because of energy considerations) at the first steps of the reaction, where oxygen is being released from U_3_O_8_, to form the UO_2_ and the enriched H_2_O. Our experimental results show depletion values relative to the starting materials, which are further supported by the theoretically calculated Δ^18^O at 600 °C, published by Hattori and Halas^19^ and Yong-fei^20^ (−6.4 and −8‰, respectively).

The data in Table 2 also allow estimating the isotopic difference between the initial U_3_O_8_ and the final UO_2_ (eq 6).
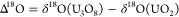
6

The average Δ^18^O value is 2.89 ± 0.82‰ (*n* = 3), suggesting that the UO_2_ products are depleted by ∼ 2.89‰ relative to the initial U_3_O_8_ at 600 °C.

## 5 Conclusions

Three U_3_O_8_ samples with different starting δ^18^O values were reduced to UO_2_ under a high-purity H_2_ atmosphere at a temperature range of 500–700 °C for 2, 4, and 6 h. We find that the final δ^18^O of UO_2_ prepared from U_3_O_8_ is determined by the reaction of the formed H_2_O and the synthesized UO_2_. The system UO_2_–H_2_O reaches isotope equilibrium at 600 and 700 °C in the longer reduction times (≥4 h). UO_2_ products are isotopically depleted relative to the U_3_O_8_ from which they were formed by 2.89 ± 0.82‰ at 600 °C. The H_2_O formed during the reaction is enriched by about 11‰ relative to the UO_2_ due to the uranium mass effect. In nuclear forensic investigations, a crucial aspect revolves around understanding the relationship between materials seized outside of regulatory control. Hence, our findings can serve as an additional tool to shed light on the fabrication process and facilitate material linkage when U_3_O_8_ and UO_2_ are present on the scene. However, additional research is required to comprehensively comprehend the mechanisms underlying the observed isotope fractionation and to fully exploit this technique in the field of nuclear forensics.
